# Supplementary material for: Development and evaluation of a rapid visual loop-mediated isothermal amplification assay for the tcdA gene in Clostridioides difficile detection
Source: PeerJ. 2024 Aug 30;12:e17776. doi: 10.7717/peerj.17776 (PMC11368091; doi:10.7717/peerj.17776)
Supplement: Supplemental Information 1 [file peerj-12-17776-s001.doc]

**Development and Clinical Application of a Rapid and Visual Detection of *tcdA* gene in *Clostridioides difficile* by a Specific and Sensitive Loop-Mediated Isothermal Amplification Assay**

Minyi Lin^1,#^, Pu Wang^3,#^, Bingyun Lu^2^, Ming Jin^2^ , Jiasheng Tan^4^, Wei Liu^5^ , Jing Yuan^5^, Xiaomou Peng^1,*^ and Ye Chen^2,*^


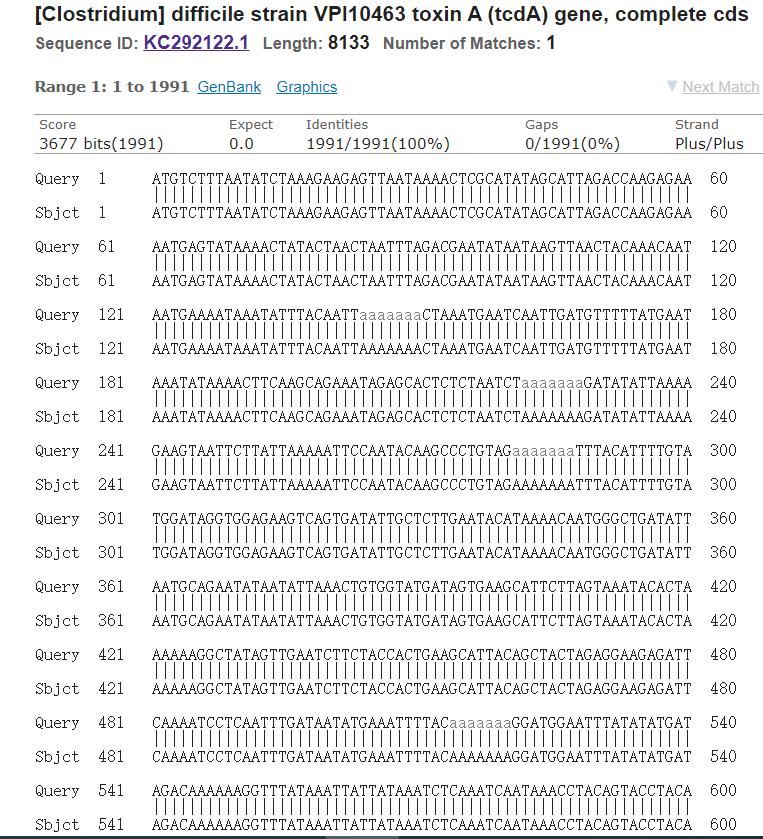

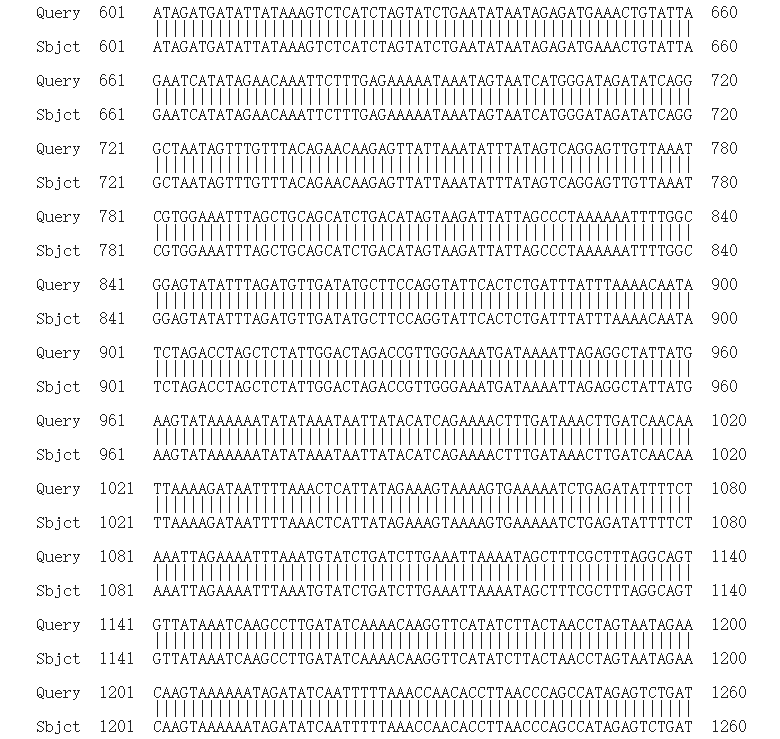


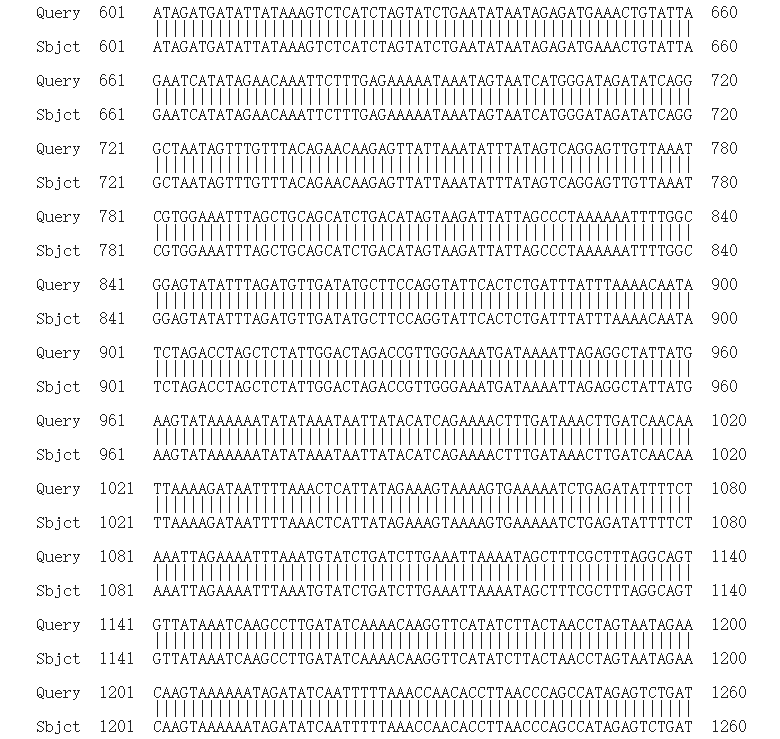


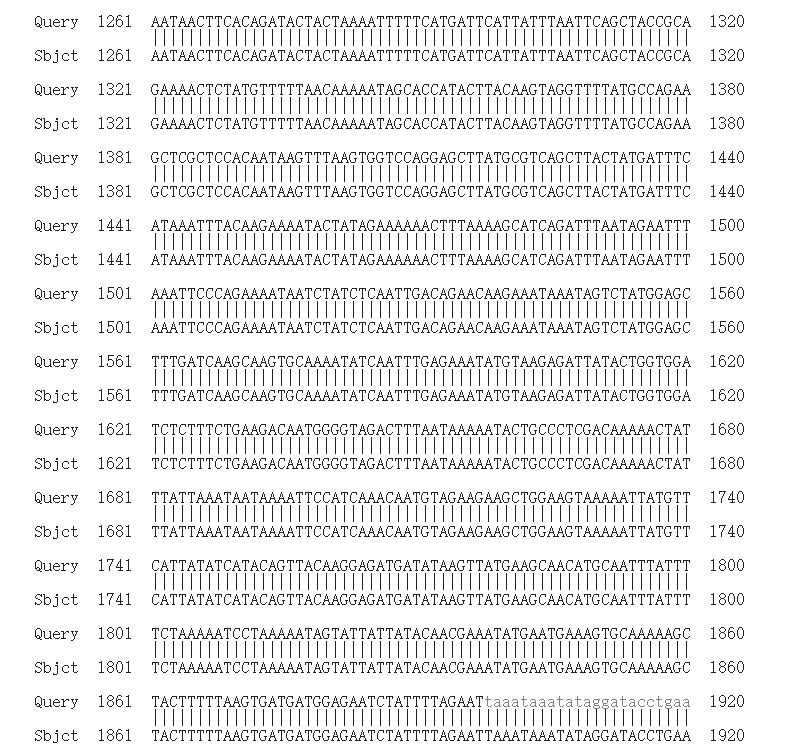


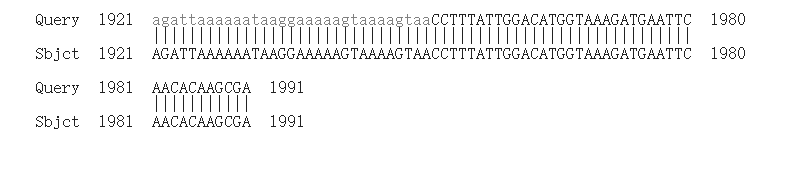


**Figure S1:** Sequence comparison of *tcdA* gene carried by *Clostridioides difficile* VPI10463 with the reported *tcdA* gene in GenBank.
