# Supplementary material for: Development and evaluation of a rapid visual loop-mediated isothermal amplification assay for the tcdA gene in Clostridioides difficile detection
Source: PeerJ. 2024 Aug 30;12:e17776. doi: 10.7717/peerj.17776 (PMC11368091; doi:10.7717/peerj.17776)
Supplement: Supplemental Information 2 [file peerj-12-17776-s002.doc]

STROBE Statement—Checklist of items that should be included in reports of ***cross-sectional studies***

|  | Item No | Recommendation | Place of appearance |
| --- | --- | --- | --- |
| **Title and abstract** | 1 | (*a*) Indicate the study’s design with a commonly used term in the title or the abstract | Methods section,lines 42-46. |
|  |  | (*b*) Provide in the abstract an informative and balanced summary of what was done and what was found | Abstract section, lines 37-58.. |
| Introduction | | |  |
| Background/rationale | 2 | Explain the scientific background and rationale for the investigation being reported | Introduction section, lines 63-118. |
| Objectives | 3 | State specific objectives, including any prespecified hypotheses | Introduction section, lines 115-118. |
| Methods | | |  |
| Study design | 4 | Present key elements of study design early in the paper | Materials & Methods section, lines120-213. |
| .Setting | 5 | Describe the setting, locations, and relevant dates, including periods of recruitment, exposure, follow-up, and data collection | Clinical stool specimens section, lines 128-145. |
| Participants | 6 | (*a*) Give the eligibility criteria, and the sources and methods of selection of participants | Clinical stool specimens section, lines 128-145. |
| Variables | 7 | Clearly define all outcomes, exposures, predictors, potential confounders, and effect modifiers. Give diagnostic criteria, if applicable | Clinical stool specimens section, lines 128-145. |
| Data sources/ measurement | 8* | For each variable of interest, give sources of data and details of methods of assessment (measurement). Describe comparability of assessment methods if there is more than one group | Materials & Methods section, lines120-213. |
| Bias | 9 | Describe any efforts to address potential sources of bias | Materials & Methods section,lines 182, 200, 207. |
| Study size | 10 | Explain how the study size was arrived at | Statistical analysis section, lines 204-208. |
| Quantitative variables | 11 | Explain how quantitative variables were handled in the analyses. If applicable, describe which groupings were chosen and why | Not applicable. |
| Statistical methods | 12 | (*a*) Describe all statistical methods, including those used to control for confounding | Statistical analysis section, lines203-213. |
|  |  | (*b*) Describe any methods used to examine subgroups and interactions | Not applicable. |
|  |  | (*c*) Explain how missing data were addressed | Statistical analysis section, lines 208-209. |
|  |  | (*d*) If applicable, describe analytical methods taking account of sampling strategy | Not applicable. |
|  |  | (*e*) Describe any sensitivity analyses | Statistical analysis section, lines203-213. |
| Results | | |  |
| Participants | 13* | (a) Report numbers of individuals at each stage of study—eg numbers potentially eligible, examined for eligibility, confirmed eligible, included in the study, completing follow-up, and analysed | Evaluation of the assay with stool specimens section, lines257-274. |
|  |  | (b) Give reasons for non-participation at each stage | Evaluation of the assay with stool specimens section, lines 259-261. |
|  |  | (c) Consider use of a flow diagram | Not applicable. |
| Descriptive data | 14* | (a) Give characteristics of study participants (eg demographic, clinical, social) and information on exposures and potential confounders | Evaluation of the assay with stool specimens section, lines 262-267. |
|  |  | (b) Indicate number of participants with missing data for each variable of interest | Evaluation of the assay with stool specimens section, lines 259-261. |
| Outcome data | 15* | Report numbers of outcome events or summary measures | Evaluation of the assay with stool specimens section, lines 267-274. |
| Main results | 16 | (*a*) Give unadjusted estimates and, if applicable, confounder-adjusted estimates and their precision (eg, 95% confidence interval). Make clear which confounders were adjusted for and why they were included | Not applicable. |
|  |  | (*b*) Report category boundaries when continuous variables were categorized | Not applicable. |
|  |  | (*c*) If relevant, consider translating estimates of relative risk into absolute risk for a meaningful time period | Not applicable. |
| Other analyses | 17 | Report other analyses done—eg analyses of subgroups and interactions, and sensitivity analyses | Not applicable. |
| Discussion | | |  |
| Key results | 18 | Summarise key results with reference to study objectives | Discussion section, lines 289-305. |
| Limitations | 19 | Discuss limitations of the study, taking into account sources of potential bias or imprecision. Discuss both direction and magnitude of any potential bias | Discussion section, lines 322-331. |
| Interpretation | 20 | Give a cautious overall interpretation of results considering objectives, limitations, multiplicity of analyses, results from similar studies, and other relevant evidence | Discussion section, lines276-331. |
| Generalisability | 21 | Discuss the generalisability (external validity) of the study results | Discussion section, lines 306-321. |
| Other information | | |  |
| Funding | 22 | Give the source of funding and the role of the funders for the present study and, if applicable, for the original study on which the present article is based | Funding statement section. |

*Give information separately for exposed and unexposed groups.

**Note:** An Explanation and Elaboration article discusses each checklist item and gives methodological background and published examples of transparent reporting. The STROBE checklist is best used in conjunction with this article (freely available on the Web sites of PLoS Medicine at http://www.plosmedicine.org/, Annals of Internal Medicine at http://www.annals.org/, and Epidemiology at http://www.epidem.com/). Information on the STROBE Initiative is available at www.strobe-statement.org.
