# Supplementary material for: Development and evaluation of a rapid visual loop-mediated isothermal amplification assay for the tcdA gene in Clostridioides difficile detection
Source: PeerJ. 2024 Aug 30;12:e17776. doi: 10.7717/peerj.17776 (PMC11368091; doi:10.7717/peerj.17776)
Supplement: Supplemental Information 3 [file peerj-12-17776-s003.zip › tcdA-Raw data/Fig4B.doc]

**Original images for figure 4B**
