# Supplementary material for: Development and evaluation of a rapid visual loop-mediated isothermal amplification assay for the tcdA gene in Clostridioides difficile detection
Source: PeerJ. 2024 Aug 30;12:e17776. doi: 10.7717/peerj.17776 (PMC11368091; doi:10.7717/peerj.17776)
Supplement: Supplemental Information 3 [file peerj-12-17776-s003.zip › tcdA-Raw data/Table3/Original images for gels of PCR in Table 3.doc]

## **Original images for gels of PCR in Table 3**

**1.The PCR results of *tcdA* gene detection were 1 positive and 16 negative.**

**2.The PCR results of *tcdA* gene detection were 2 positive and 20 negative.**

3. **The PCR results of *tcdA* gene detection were 2 positive and 25 negative.**

4. **The PCR results of *tcdA* gene detection were 3 positive and 14 negative.**

5. **The PCR results of *tcdA* gene detection were 4 positive and 23 negative.**

6. **The PCR results of *tcdA* gene detection were 4 positive and 50 negative.**
