# Supplementary material for: Development and evaluation of a rapid visual loop-mediated isothermal amplification assay for the tcdA gene in Clostridioides difficile detection
Source: PeerJ. 2024 Aug 30;12:e17776. doi: 10.7717/peerj.17776 (PMC11368091; doi:10.7717/peerj.17776)
Supplement: Supplemental Information 3 [file peerj-12-17776-s003.zip › tcdA-Raw data/Table3/Sequencing results of 18 tcdA genes in Table 3.doc]

## **Sequencing results of 18 *tcdA* genes in Table 3**

1. **No. 7 *tcdA* gene sequencing results of LAMP positive PCR negative specimens.**

2. **No. 8 *tcdA* gene sequencing results of LAMP positive PCR negative specimens.**

3. **No. 10 *tcdA* gene sequencing results of LAMP positive PCR negative specimens.**

4. **No. 11 *tcdA* gene sequencing results of LAMP positive PCR negative specimens.**

5. **No. 15 *tcdA* gene sequencing results of LAMP positive PCR negative specimens.**

6. **No. 27 *tcdA* gene sequencing results of LAMP positive PCR negative specimens.**

7. **No. 28 *tcdA* gene sequencing results of LAMP positive PCR negative specimens.**

8. **No. 34 *tcdA* gene sequencing results of LAMP positive PCR negative specimens.**

9. **No. 37 *tcdA* gene sequencing results of LAMP positive PCR negative specimens.**

10. **No. 38 *tcdA* gene sequencing results of LAMP positive PCR negative specimens.**

11. **No. 44 *tcdA* gene sequencing results of LAMP positive PCR negative specimens.**

12. **No. 62 *tcdA* gene sequencing results of LAMP positive PCR negative specimens.**

13. **No. 81 *tcdA* gene sequencing results of LAMP positive PCR negative specimens.**

14. **No. 92 *tcdA* gene sequencing results of LAMP positive PCR negative specimens.**

15. **No. 165 *tcdA* gene sequencing results of LAMP positive PCR negative specimens.**

16. **No. 6 *tcdA* gene sequencing results of LAMP negative PCR positive specimens.**

17. **No. 75 *tcdA* gene sequencing results of LAMP negative PCR positive specimens.**

18. **No. 76 *tcdA* gene sequencing results of LAMP negative PCR positive specimens.**
